# Analgesic Effect of Dexmedetomidine-Nalbuphine Combination vs. Dexmedetomidine Alone in Donkeys Undergoing Field Castration under Total Intravenous Anesthesia

**DOI:** 10.3390/ani14172452

**Published:** 2024-08-23

**Authors:** Ibrahim E. Helal, Hatim A. Al-Abbadi, Mohamed A. Hashem, Heba M. A. Abdelrazek, Mohammed H. Shekidef, Mahmoud F. Ahmed

**Affiliations:** 1 Department of Agriculture, Faculty of Environmental Science, King Abdulaziz University, Jeddah 80208, Saudi Arabia; ibrahim_hilal@vet.suez.edu.eg; 2 Department of Surgery, Anesthesiology and Radiology, Faculty of Veterinary Medicine, Suez Canal University, 4.5 Ring Road, Ismailia 41522, Egypt; mohamed.hashem@vet.suez.edu.eg (M.A.H.); shekidef77@vet.suez.edu.eg (M.H.S.); 3 Faculty of Medicine, University Hospital, King Abdulaziz University, Jeddah 80212, Saudi Arabia; hatimalabbadi@yahoo.com; 4 Department of Physiology, Faculty of Veterinary Medicine, Suez Canal University, 4.5 Ring Road, Ismailia 41522, Egypt; heba_abdelrazek@vet.suez.edu.eg

**Keywords:** analgesia, castration, dexmedetomidine, donkey, ketamine, nalbuphine, propofol, total intravenous anesthesia

## Abstract

**Simple Summary:**

Donkey welfare and pain relief are of particular importance in the veterinary field. Therefore, pain management during surgical operations in field settings is mandatory. The current study sheds light on the antinociceptive effect of a combination of dexmedetomidine and nalbuphine premedication prior to total intravenous anesthesia using ketamine-propofol in castration procedures in field conditions. Shortly after castration, the proposed combination of dexmedetomidine and nalbuphine resulted in decreased postoperative pain. In addition, the cardiac function was improved compared to using dexmedetomidine alone during anesthesia. This was demonstrated using established clinicophysiological assessments, serum biochemical markers, and behavioral pain scores for six hours post-recovery. Dexmedetomidine and nalbuphine premedication prior to total intravenous anesthesia using ketamine-propofol showed potential benefits compared to dexmedetomidine alone in providing more analgesia and managing postoperative pain in jacks undergoing castration under field conditions.

**Abstract:**

This study evaluated the antinociceptive effect of dexmedetomidine-nalbuphine vs. dexmedetomidine alone in jacks undergoing field castration under total intravenous anesthesia. Jacks were premedicated with intravenous (IV) dexmedetomidine (5 µg/kg), either alone (Group D, *n* = 6) or in combination with 0.3 mg/kg nalbuphine (Group DN, *n* = 6). IV ketamine (1.5 mg/kg) and propofol (0.5 mg/kg) were used to induce general anesthesia, which was maintained by a continuous propofol (0.2 mg/kg/min) IV infusion. The quality of anesthesia, analgesia, and recovery were evaluated. A simple descriptive scale (SDS) was used to measure pain from the recovery time to 6 h later. The DN group exhibited improvements in analgesic and recovery quality and SDS of pain at 1-, 2-, and 3-h post-recovery. There was an apparent improvement in cardiac status, as evidenced by the enhanced heart rate and electrocardiogram findings compared to group D during surgery and recovery time. The DN group had a lower level of inflammatory cytokines, both during the surgery and shortly after recovery. Therefore, the dexmedetomidine-nalbuphine combination prior to IV anesthesia of ketamine and propofol in jacks undergoing field castration resulted in a stable surgical plane of anesthesia, improved antinociception, less pain postoperatively, and better cardiac stability.

## 1. Introduction

Donkey welfare and pain relief are of particular interest because of their importance as a workforce in several countries [1,2]. The approach to pain management in donkeys requires the use of suitable analgesic drugs. Orchiectomy, a husbandry practice, is known to cause stressful conditions and evoke pain that negatively influences the well-being of donkeys and their immunological response [3,4]. For surgical interventions that need general anesthesia, it is recommended to use total intravenous anesthesia (TIVA) in field settings. Protocols of TIVA are preferred in equines because they are more cost-effective, particularly in developing countries than inhalation anesthesia [5,6]. Therefore, there is an innate need for drugs that have minimal side effects yet nevertheless provide effective antinociception and postoperative pain relief [7,8].

Dexmedetomidine is the most selective for the α2-adrenergic receptor and the most potent member of the α2-agonists available [9]. It is frequently used in conjunction with opioids to improve the quality of sedation and analgesia [10]. The cardiopulmonary action is comparable to that of other α2-adrenergic receptor agonists, characterized by bradycardia, a decrease in cardiac output, and an initial elevation in systemic arterial blood pressure (BP) followed by hypotension [11].

The development of drugs with selective opiate receptor activation has resulted in improved analgesia while minimizing respiratory depression and excitatory effects. Nalbuphine is a semi-synthetic narcotic κ agonist and μ antagonist analgesic [12]. It is used to overcome opioid-induced cardiopulmonary depression and sustain κ-mediated analgesia [12,13]. Therefore, nalbuphine is proposed as an appealing narcotic analgesic with fewer undesirable effects and decreased regulatory limitations than other opioids.

This study hypothesized that the combination of dexmedetomidine and nalbuphine prior to TIVA would provide a more antinociceptive effect during surgery, provide more postoperative pain relief, and minimize the adverse effect on cardiac function of donkeys undergoing field orchiectomy compared to using dexmedetomidine alone.

## 2. Materials and Methods

This study was conducted in compliance with the Institutional Animal Use and Care Committee of the Faculty of Veterinary Medicine at Suez Canal University, which reviewed and authorized all experimental procedures (Approval Number SCU2024006). The study was carried out as a randomized, blinded prospective clinical study.

### 2.1. Donkeys

The study was conducted on twelve Egyptian Baladi male donkeys who were scheduled for bilateral orchiectomy at the Teaching Farm of the Faculty of Veterinary Medicine at Suez Canal University. Jacks were assigned randomly into two groups: dexmedetomidine (Group D), which served as a control group (*n* = 6), or dexmedetomidine-nalbuphine (Group DN) (*n* = 6). There were no statistical differences in age (Group D: 29.3 ± 10.3 months; Group DN: 28.7 ± 9.6 months) or body weight (Group D: 121.5 ± 24.5 kg; Group DN: 129.2 ± 19.6 kg) found between the two groups. The sample size calculation was performed using G*Power version 3.1.9.2 [14]. The effect size convention d was 1.6 using an alpha (α) level of 0.05 and a beta (β) level of 0.05, i.e., power = 80%. The study was conducted on healthy jacks based on the general physical examination, electrocardiography (ECG), and findings of the complete blood count (CBC) and serum biochemical analyses. All Jacks had normal-descended testes; no cryptorchids were used in the study. Food, but not water, was withheld for 8 h prior to anesthesia.

### 2.2. Anesthesia and Surgical Protocol

A 14-gauge catheter was inserted aseptically into the left external jugular vein for drug delivery, while another catheter was placed in the right external jugular vein for the collection of venous blood samples. Dexmedetomidine HCl (Precedex^®^; Hospira Inc., Rocky Mount, NC, USA) was administered intravenously (IV) at a dose of 5 µg/kg [15] 10 min before the induction of general anesthesia. Nalbuphine HCl (Nalufin^®^; Amoun Pharmaceutical Company, Al Qalyubia, Egypt) was given IV to the DN group at a dose of 0.3 mg/kg [16] five minutes after the bolus dose of dexmedetomidine. Before TIVA, sedation quality was assessed using a basic descriptive scale (0–3) developed by Lizarraga et al. [15] (Appendix A). Anesthesia was induced in a padded box via the IV injection of ketamine HCl (KETAMAX-50; Troikaa Pharmaceuticals Ltd., Uttarakhand, India) at a dose of 1.5 mg/kg, followed immediately by the IV propofol injection at a dose of 0.5 mg/kg (Diprivan^®^, AstraZeneca, Macclesfield, UK) [17]. The jacks were placed in a right lateral recumbent position on a thick rubber mattress, and a tracheal tube was inserted. Anesthesia was maintained with a continuous-rate infusion (CRI) of propofol at 0.2 mg/kg/minute [6], and jacks were breathing atmospheric air throughout the procedure. After aseptic preparation of the surgical field, castration was performed by the closed method via the scrotal approach by applying a transfixing suture around the spermatic cord using 2 absorbable polyglycolic acid (EGYSORB; Taisier Group, Cairo, Egypt) before transection, and the surgical wound was left to heal by secondary intention. The same surgeon performed the bilateral orchiectomy throughout the study. After the end of the castration procedure, propofol infusion ceased, and jacks were allowed to recover unassisted.

### 2.3. Cardiorespiratory and Body Temperature Measurements

All Jacks were monitored, including the recording of heart rate (HR) by auscultation, respiratory rate (RR) by counting thoracolumbar expansion, pulse rate, and oxygen saturation (SpO_2_) by pulse oximeter (Generra 530, Generra Medical Inc., Clearwater, FL, USA) through the tongue. Systolic and diastolic blood pressures were recorded using non-invasive BP measurements using an automatic BP monitor (Granzia LD-562, Honsun Co. Ltd., Shanghai, China) through a cuff placed over the left brachial artery. ECG examination was performed using (Contec ECG machine 3 channel 300GA, Qinhuangdao, China) by applying the base-apex method as described by da Costa et al. [18], where the device paper speed was set to be 25 mm/s and sensitivity was 10 mm = 1 mV. Leads I, II, and III were recorded. Meanwhile, rectal temperature (RT) was measured by a rectal thermometer. The physiological parameters, including RT, RR, and BP, were recorded before administration of drugs (baseline), 10 min after premedication T (pre), as well as at 5, 15, and 30 min after the induction of general anesthesia (T5, T15, and T30, respectively), and when the jacks stood T (recovery), while HR and ECG were recorded at the same time points in addition to ½ hour after recovery (PR½). Meanwhile, pulse rate and SpO_2_ were recorded at T5, T15, T30, and T recovery, respectively.

### 2.4. Anesthetic Monitoring and Data Collection

The time for induction (in seconds) was measured for each jack. The quality of induction was scored (1–3) [17]. The quality of analgesia (response to surgical stimulation) scored (1–4) [19], while muscle relaxation scored (0–3) [20] and palpebral reflex scored (0–2) [4]. The duration of anesthesia from induction to the end of TIVA (in minutes), the surgical time from skin incision to complete transection of both testicles (in minutes), and the total recovery time (in minutes), including time to sternal recumbency (in minutes) and time to stand (in minutes) were recorded. The anesthetic maintenance scored (0–3) [21], and the quality of recovery scored (1–5) [22] (Appendix A).

### 2.5. Hematological and Serum Biochemical Analyses

Baseline blood samples were collected before administration of dexmedetomidine (baseline), 10 min following premedication T (pre), 5, 15, and 30 min following the induction of general anesthesia (T5, T15, and T30, respectively), when the jacks stood T (recovery), and ½, 1, 2, 4, and 6 h after recovery (PR½, PR1, PR2, PR4, and PR6, respectively). Jugular blood samples were obtained from a fixed cannula into plain, sodium fluoride, and ethylene diamine tetra-acetic acid (EDTA) tubes. Sera were separated from plain tubes after clotting and centrifugation at 3000 rpm for 15 min. Sera were kept at −80 °C until analysis. Fluorinated plasma was immediately separated at 3000 rpm for 15 min.

The EDTA blood tubes were immediately submitted to a normal hematological examination for red blood cells (RBCs) and white blood cells (WBCs) using a modified Neubauer chamber. Serum interleukin-6 (IL-6) and tumor necrosis factor-alpha (TNF-α) were estimated using ELISA assay kits according to manufacturer instructions (Cat. No. CSB-E16634Hs and CSB-E14031Hs, Cusabio Technology LLC, Houston, TX, USA), respectively. Horse Cardiac Troponin I (cTn-I) was detected in sera using ELISA assay kits according to manufacturer instructions (Cat. No. MBS017380, MyBioSource, San Diego, CA, USA). Serum alanine aminotransferase (ALT) and aspartate aminotransferase (AST) were determined using calorimetric kits (SPINREACT, Girona, Spain) at a wavelength of 340 nm. Serum creatinine was estimated using a calorimetric kit (SPINREACT, Girona, Spain) at a wavelength of 492 nm. Immediately separated fluorinated plasma was subjected to glucose estimation using an enzymatic glucose kit (Cat. No. S1144A, SPINREACT, Girona, Spain).

### 2.6. Pain Assessment

An experienced investigator blinded to the study groups had evaluated and recorded the degree of pain before premedication at baseline and at 1, 2, 3, 4, and 6 h after recovery using a simple descriptive scale (SDS) for pain as described by Love et al. [23] (Appendix A).

### 2.7. Statistical Analysis

The normal distribution was estimated using the Shapiro-Wilk test. Descriptive statistics were calculated in the form of the mean ± standard deviation (SD). For parametric variables, an independent student’s *t*-test was performed to compare the two groups in terms of age, body weight, duration of anesthesia, surgical time, time to sternal recumbency, and total recovery time. For non-parametric variables, descriptive statistics were calculated in the form of the median ± interquartile range (IQR). The Mann-Whitney test was performed to compare the two groups in terms of quality of induction, quality of analgesia, muscle relaxation, palpebral reflex, anesthetic maintenance, and quality of recovery.

A linear mixed-effects model was used to analyze the impact of the group and time on the dependent variables. The model was fitted in R using the lme4 package [24]. Subjects were included as a random effect in the model to account for repeated measurements within subjects. For each dependent variable, we fitted separate models using the restricted maximum likelihood (REML) criterion. Post-Hoc Test Following the model fitting, the emmeans package [25] was used to calculate the estimated marginal means for the time periods across the groups. Statistical analysis was performed using R software [26]. A *p*-value < 0.05 is considered statistically significant. The graphs were created using GraphPad Prism software version 8.0.2 (GraphPad Software, Inc., Boston, MA, USA).

## 3. Results

### 3.1. Evaluation of Sedation, Anesthetic Induction, Maintenance and Recovery

Before induction of general anesthesia, all Jacks in both groups exhibited varying degrees of sedation, ranging from moderate to marked. However, only one Jack in the D group showed mild sedation. After the injection of nalbuphine in the DN group, the sedation depth increased further, resulting in a significant increase in the quality and depth of sedation compared to the D group. Jacks in both groups showed a quick induction onset of less than one minute after IV injections of ketamine and propofol, with no significant difference between the D and DN groups. A good to acceptable induction quality without paddling or progressive movement was observed, with no notable difference between the D and DN groups. In both groups, there were no complications during the castration procedure, and the surgical time was relatively short in all jacks, ranging from 14 to 21 min. The quality of analgesia was remarkably enhanced in the DN group, although the muscular relaxation score and palpebral reflex did not differ substantially between the D and DN groups. The anesthetic maintenance ranged from good to excellent, and no Jacks required additional propofol bolus doses without variation between the D and DN groups. The duration of anesthesia, from induction to the endpoint of TIVA, did not differ significantly between the D and DN groups. However, Jacks of the DN group showed a significant delay in terms of time to sternal recumbency and total recovery time, respectively. In both groups, there was a smooth recovery from anesthesia; the degree of recovery ranged from excellent to good in the DN group, while the D group ranged from good to fair, with a significant difference between the D and DN groups (Table 1 and Table 2).

### 3.2. The Findings of Cardiorespiratory Measurements

**Figure 1:**
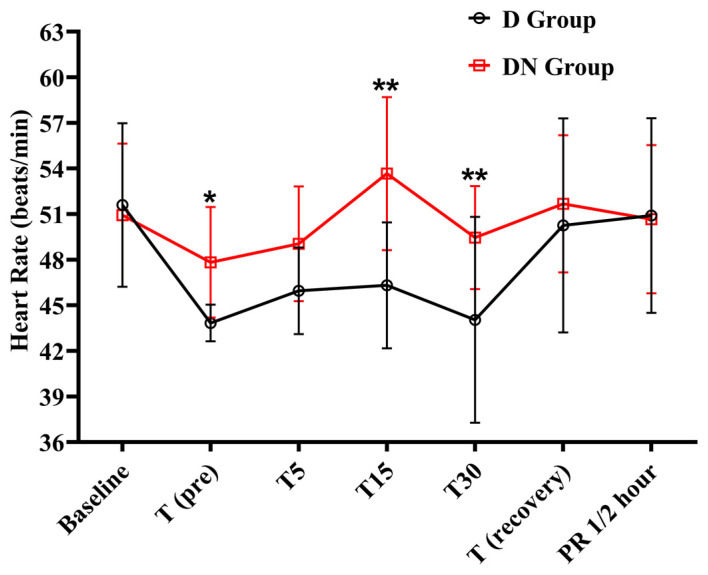
Heart rate changes for jacks undergoing surgical castration under the effects of dexmedetomidine (D group) or dexmedetomidine-nalbuphine combination (DN group). T (pre): 10 min following premedication; T5, T15, and T30: 5, 15, and 30 min following the induction of general anesthesia, respectively; T (recovery): recovery time; and PR½: ½ hour after recovery. Data were expressed as mean ± SD. * indicates *p* < 0.05, while ** indicates *p* < 0.01 between the groups.

Bradycardia was observed at the T (pre), T5, T15, and T30 time points in the D group, as compared to the baseline. Following that, there was a steady rise toward baseline values during the recovery period. Similarly, the DN group also showed bradycardia at the T (pre) and T5 time points. The DN group had significantly higher HR at T (pre), T15, and T30 compared to the D group (Figure 1 and Table 3). Similarly, the pulse rate followed the same trend as the HR.

**Figure 2:**
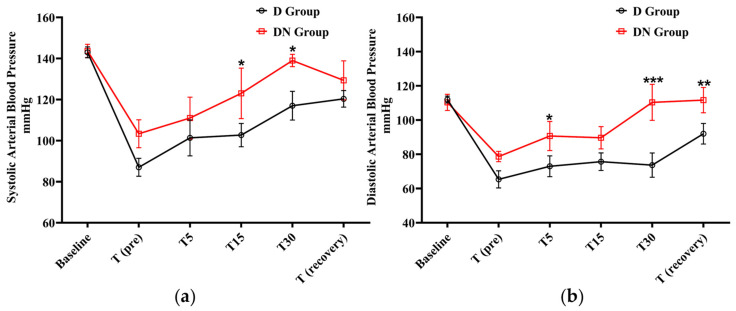
Blood pressure (BP) changes for jacks undergoing surgical castration under the effects of dexmedetomidine (D group) or the dexmedetomidine-nalbuphine combination (DN group). (**a**) Systolic arterial BP, and (**b**) Diastolic arterial BP. T (pre): 10 min following premedication; T5, T15, and T30: 5, 15, and 30 min following the induction of general anesthesia, respectively; and T (recovery): recovery time. Data were expressed as mean ± SD. * indicates *p* < 0.05, ** indicates *p* < 0.01, and *** indicates *p* < 0.001 between the groups.

After premedication administration, a decrease in systolic and diastolic BP was observed in the D and DN groups. The D group showed significantly lower systolic BP at T15 and T30 in comparison to the DN group. In the same pattern, the diastolic BP was significantly lower in the D group at T5, T30, and T (recovery) compared to the DN group. The administration of nalbuphine resulted in higher systolic and diastolic BP compared to the D group at all the tested time points (Figure 2 and Table 4).

The ECG measures, including PR, QT, and QRS intervals, showed prolongation at the tested time points when compared to baseline values in both groups. The duration was higher in the D group compared to the DN group. In contrast, the ST segment deviation did not change significantly across the examined time points in both groups compared to baseline values, except for T (recovery). The findings revealed a significant increase in the PR interval among jacks in the D group compared to the DN group at T5, T15, and T (recovery), indicating possible atrioventricular conduction delays. Similarly, the QT interval was significantly higher in the D group at T (pre), T5, T15, T30, and T (recovery) compared to the DN group. Meanwhile, in the D group, the ST segment deviation showed a significant difference at T5, T15, and T (recovery) between the two groups (Table 5).

**Figure 3:**
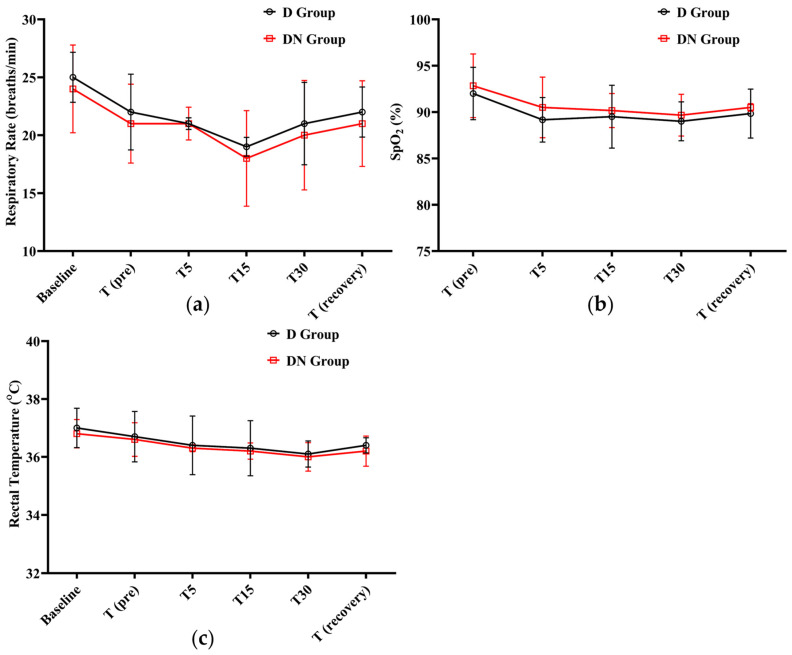
Respiratory rate (RR), oxygen saturation (SpO_2_%), and rectal temperature (RT) changes for Jacks undergoing surgical castration under the effects of dexmedetomidine (D group) or the dexmedetomidine-nalbuphine combination (DN group) at different time points. (**a**) RR; (**b**) SpO_2_%; and (**c**) RT. T (pre): 10 min following premedication; T5, T15, and T30: 5, 15, and 30 min following the induction of general anesthesia, respectively; and T (recovery): recovery time. Data were expressed as mean ± SD.

After administering premedication, the RR steadily decreased. The lowest RR was recorded at T15 (19 ± 0.81, 18 ± 4.12) in the D and DN groups, respectively. A gradual increase was observed until the recovery period. Meanwhile, there was no significant difference between the DN group and the D group at any given time. In both groups, SpO_2_% did not fall significantly from the baseline; the lowest SpO_2_% recorded was 89 ± 2.1 at T30 in the D group. Additionally, no clinical complications were observed, and oxygen supplementation was not required. Furthermore, RT decreased in both groups, but there was no significant difference between them at the time points tested (Figure 3 and Table 6).

### 3.3. The Findings of CBC and Serum Biochemical Analyses

The CBC results revealed no significant variations in RBC count, hemoglobin (Hb), or packed cell volume (PCV)% between the D and DN groups or between the two groups and the baseline values (Table S1). In contrast, the WBC count increased in both groups, from T15 to PR 6. The DN group showed a non-significant overall effect on WBC count. Both the neutrophil count and the neutrophil-lymphocyte ratio (NLR) followed the same pattern as the WBC count. Starting with T15, an increase in neutrophils and NLR was observed, but the DN group showed a significant decrease in neutrophil and NLR values compared to the D group from T30 to PR 6 (Table 7). The overall effect of the DN group on lymphocyte counts was not significant.

**Figure 4:**
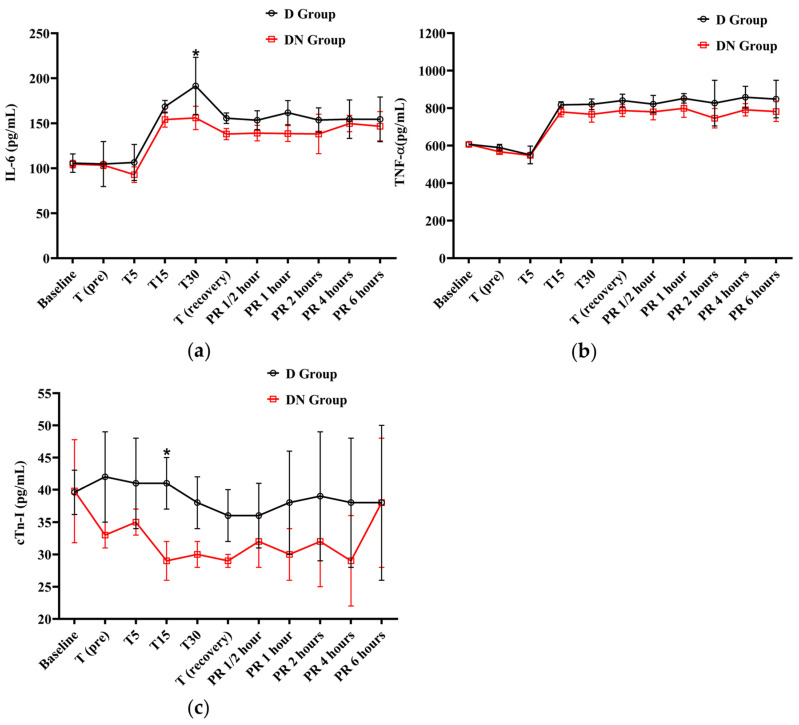
The findings of interleukin-6 (IL-6), tumor necrosis factor-alpha (TNF-α), and cardiac troponin I (cTn-I) for Jacks undergoing surgical castration under the effects of dexmedetomidine (D group) or the dexmedetomidine-nalbuphine combination (DN group). (**a**) IL-6, (**b**) TNF-α, and (**c**) cTn-I. T (pre): 10 min following premedication; T5, T15, and T30: 5, 15, and 30 min following the induction of general anesthesia, respectively. T (recovery): recovery time; PR½: ½ hour after recovery; PR 1: 1 h after recovery; PR 2: 2 h after recovery; PR 4: 4 h after recovery; PR 6: 6 h after recovery. Data were expressed as mean ± SD. * indicates *p* < 0.05 between the groups.

The IL-6 levels were elevated from T15 to PR 6, surpassing the baseline levels in both groups. At T30, the D group had a significant increase in IL-6 levels compared to the DN group. Similarly, the levels of TNF-α showed an increase from T15 to PR 6 in both groups when compared to the baseline. The D group showed higher TNF-α levels than the D group, but there was no significant group interaction found, as shown in Figure 4a,b and Table 8.

**Figure 5:**
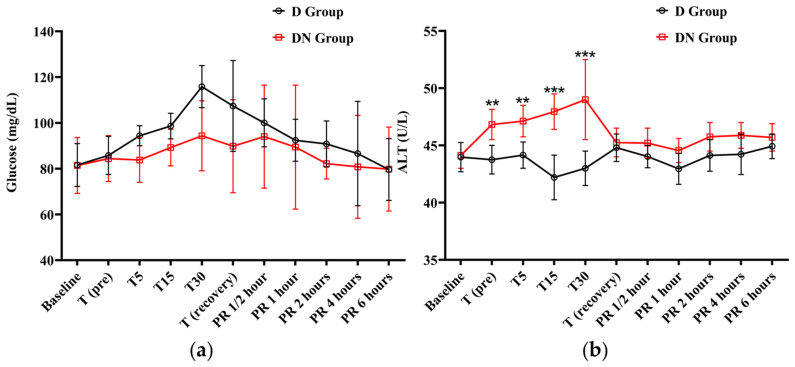
The findings of blood glucose level and alanine transaminase (ALT) for Jacks undergoing surgical castration under the effects of dexmedetomidine (D group) or the dexmedetomidine-nalbuphine combination (DN group) at different time points. (**a**) Blood glucose level and (**b**) ALT. T (pre): 10 min following premedication; T5, T15, and T30: 5, 15, and 30 min following the induction of general anesthesia, respectively; T (recovery): recovery time; PR½: ½ hour after recovery; PR 1: 1 h after recovery; PR 2: 2 h after recovery; PR 4: 4 h after recovery; PR 6: 6 h after recovery. Data were expressed as mean ± SD. ** indicates *p* < 0.01, while *** indicates *p* < 0.001 between the groups.

The cTn-I values did not show a significant variation compared to baseline values in the D and DN groups. The levels of cTn-I were higher in the D group compared to the DN group, and there was a significant increase at T15 (Figure 4c and Table 8). Hyperglycemia was reported in both groups following premedication administration. The peak of hyperglycemia was recorded at T30 and T (recovery), followed by a gradual decrease to the baseline values at PR 4. The DN group showed a lower blood glucose level than the D group, but no significant group interaction was found, as shown in Figure 5a and Table 9. 

The ALT levels only increased in the DN group at T (pre), T5, T15, and T30. There was a significant difference between the two groups at these time points. After that, the ALT level gradually decreased to the baseline values at recovery time. These findings suggest that the nalbuphine administration affects ALT levels over time (Figure 5b and Table 9). Meanwhile, both AST and creatinine demonstrated no statistically significant main effects or interactions, indicating that the changes in AST and creatinine over time did not differ significantly between the two groups (Table S2).

### 3.4. Evaluation of Pain after Recovery from TIVA Using the SDS for Pain

**Figure 6:**
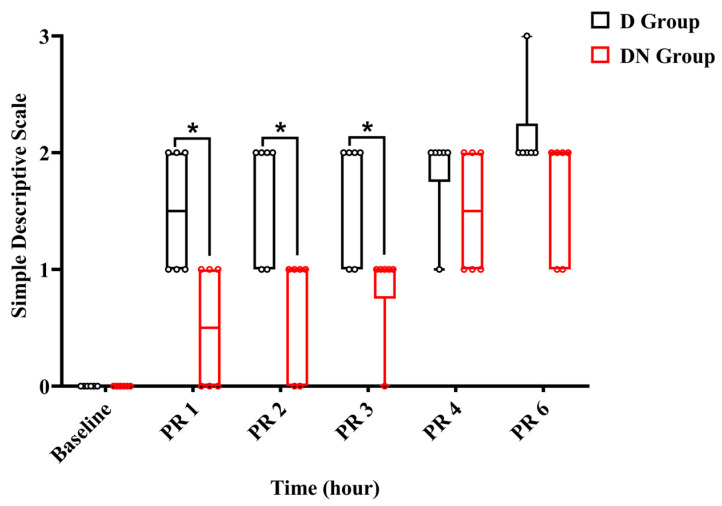
A boxplot graph showed a simple descriptive scale (SDS) in the D and DN groups at baseline and 1, 2, 3, 4, and 6 h after recovery. The box represents the min-to-max of the data, with the horizontal line within indicating the median. PR 1: 1 h after recovery; PR 2: 2 h after recovery; PR 4: 4 h after recovery; PR 6: 6 h after recovery. * Indicates *p* < 0.05 between the groups.

Throughout the observation period, the jacks were either standing or moving inside the stall. Within the first two hours after recovery, jacks in the DN group had scores of (0–1), indicating a state of alertness and interaction, while others showed signs of being subdued and apprehensive during interactions. Meanwhile, in the D group, the jacks scored (1–2). Throughout the interaction, two jacks demonstrated a subdued and anxious attitude, whereas four jacks were walking stiffly and not interacting willingly. Three hours after recovery, five jacks in group DN scored 1, and one donkey scored 0. In contrast, four jacks in group D achieved a score of 1, and two jacks achieved a score of 2. No jacks reached score 3 except one jack in the D group 6 h post-recovery, and rescue analgesia was immediately given using 1.1 mg/kg bodyweight of flunixin meglumine IV. The SDS pain score showed a significant increase at all time points post-recovery compared to the preoperative baseline. The interaction effects at PR 1, PR 2, and PR 3 were significant, indicating that the SDS pain score increased over time and was less pronounced in the DN group compared to group D. Meanwhile, at PR 4 and PR 6, the interaction effects were not significant, suggesting no significant difference in the change in pain scores between the two groups at these times (Figure 6 and Table 10).

## 4. Discussion

In the present study, combining α2-adrenergic agonist (dexmedetomidine) with nalbuphine as a premedication was tested to improve analgesia and maintain stable cardiac function for Jacks undergoing orchiectomy in field conditions under a TIVA protocol, including ketamine and propofol. The combination of dexmedetomidine and nalbuphine served as multimodal analgesia to manage pain by acting on different receptors along the nociceptive system. The significance of this method resides in its synergistic analgesic property, which promotes intra- and postoperative analgesia. In this study, an orchiectomy was performed to examine our multimodal analgesia model, as the pain experienced during an orchiectomy serves as a typical nociceptive stimulus [27].

Inadequate preoperative pain management might result in cardiac decompensation after anesthetic induction [28]. Prior to the procedure, nonsteroidal anti-inflammatory drugs (NSAIDs) were not administered to minimize any possible effect on the results in both groups. Nevertheless, all animals were given NSAIDs six hours after the surgery and for the next five days.

The TIVA was chosen in this study because it is a standard anesthetic approach used in equines for castration [4,5]. Several drug combinations with ketamine have been utilized for the induction and short-term maintenance of TIVA [29]. In the present study, TIVA was induced using a combination of ketamine and propofol [17]; based on an earlier report, this combination prompted superior induction and muscle relaxation with a longer anesthetic time and smoother recovery than ketamine alone [30]. Meanwhile, maintenance was performed with a CRI of propofol only [6] since donkeys metabolize ketamine faster than horses [28], and propofol was reported to provide better muscle relaxation, especially with the avoidance of ketamine in the maintenance protocol [6].

In the present study, the DN group showed more marked sedation than the D group. This could be attributed to the fact that opioids and α2 adrenergic agonists have been demonstrated to induce significant sedation and pain relief in horses, which can be related to their synergistic effects [16,29,31,32]. The DN group exhibited a significant improvement in analgesia quality, recovery quality, and increased recovery duration. These results aligned with earlier studies on horses premedicated with a xylazine-nalbuphine combination prior to TIVA [31] and donkeys premedicated with detomidine-butorphanol [4]. In addition, anesthesia had remained stable with clinically acceptable cardiorespiratory effects under CRI of propofol alone, as previously described [6]. In addition, there was no need for additional doses of propofol, which may be linked to the shorter duration of surgical time in both groups. Moreover, this study showed a rapid, smooth recovery, which may be attributed to propofol’s rapid onset of action and short half-life following short-duration procedures [6].

In this study, HR fell markedly following sedation in the D group but not in the DN group. These findings could be attributed to the central activation of the presynaptic α-2 adrenoceptors in the central nervous system (CNS) by dexmedetomidine. This activation inhibited sympathetic outflow, resulting in the observed bradycardia and hypotension [33]. Meanwhile, administration of nalbuphine (κ agonist) ameliorated dexmedetomidine-induced bradycardia and hypotension. This could be linked to the activation of κ receptors in several loci in the CNS, which in turn increased the HR [34]. Moreover, nalbuphine as a κ agonist might potentially influence renal sympathetic supply and increase sodium and potassium reabsorption, promoting peripheral vascular resistance and restoring reduced BP [35,36]. Finally, the significant increase in BP in the DN group in comparison to the D group, particularly diastolic pressure, may be attributed to the regulation of baroreflex in arteries via the κ receptor agonism of nalbuphine [37]. These findings were consistent with the findings of previous reports that showed that the nalbuphine-xylazine combination showed effective analgesic and sedative outcomes with cardiovascular stability in calves [38], camels [39], and horses [31]. Moreover, Taylor et al. [16] reported that nalbuphine had little effect on the cardiovascular system in ponies.

In the current study, the D group had significantly longer PR and QT intervals compared to the DN group. Multiple mechanisms may contribute to this, including central and peripheral presynaptic α-2 adrenoceptor agonism, which impedes sympathetic signaling, resulting in bradycardia, which probably blocks the atrioventricular node, prolonging PR and QT intervals [40,41]. On the other hand, administration of nalbuphine with dexmedetomidine significantly mitigated the prolonged PR and QT intervals at different durations. Activation of κ receptors in cardiomyocytes primes the release of Ca^2+^ [42], which mediates the automaticity and duration of SA and AV conduction [43], therefore shortening PR and QT intervals. The ST elevations exhibited greater amelioration in the DN group than in the D group. This effect is mediated via κ receptor negative modulation of β-adrenergic receptors that are implicated in myocardial ischemia, causing ST elevation via a cAMP-mediated mechanism [44]. Moreover, nalbuphine could efficiently improve the hemodynamic perturbation in myocardial infarction [45] and exert an anti-inflammatory potential via inhibiting NF-κB-induced IL-6 and TNF-α production [46]. These changes aligned with the cTn-I results, which showed significant improvement in the DN group at T15.

In the present study, the mild depression of the CNS and improved tissue perfusion by using the light level of IV general anesthesia in comparison to inhalation anesthesia may account for the absence of any statistically significant variation in RR, SpO_2_, and RT between the D group and DN group [4,6]. Moreover, several studies have shown that the respiratory effect of propofol is minimized when ketamine is administered in conjunction with propofol or with propofol together with α2-agonists [17,20].

There was a notable difference between the T (pre) and T5 in both groups compared to T15 regarding levels of IL-6, TNF-α, and WBC count. This difference was attributed to the surgical process, which triggered a painful stimulus and elicited a sympathetic response to the surgical stimulation. Moreover, the reaction to noxious stimulation resulted in a rise in HR and BP compared to the preceding times [4,47]. In comparing the two groups, the D group maintained a significantly higher neutrophil count and NLR than the DN group. These results indicate a potent anti-inflammatory response upon combination with nalbuphine via attenuating immune cells, NLR, and cytokines that are imperative in several mechanisms underlying pain and postoperative recovery [48]. Consequently, the levels of IL-6 and TNF-α were observed to be considerably higher in the D group compared to the DN group, and there was a significant increase in IL-6 at T30. This may be explained through the beneficial effects of nalbuphine, which improves postoperative pain relief and reduces surgical stress and pain [13,49]. Furthermore, nalbuphine exerts an additional central analgesic effect via κ receptors by reducing traumatic stress hormone secretion, cortisol, and catecholamines that inhibit neutrophilia and macrophage activation to achieve sedative, anti-sympathetic and antioxidant effects manifested by reduced TNF-α and IL-6 [50].

The anesthetic procedures did not induce significant increases in AST [17] and creatinine; however, ALT, a pure hepatic enzyme, was significantly increased in the DN group at T (pre), T5, T15, and T30. The elevated ALT may be related to the fact that nalbuphine is predominantly eliminated and metabolized by the liver, which may have induced hepatocyte stress, causing them to leak ALT into the blood [51,52]. Therefore, nalbuphine administration in cases of liver disease should be highlighted.

In the present study, the DN group experienced a substantial drop in pain scores that lasted up to three hours following recovery. This might be attributed to the antinociceptive effects of nalbuphine hydrochloride, which last for about 3 h [31,38]. Furthermore, nalbuphine was discovered to hinder the release of substance P from afferents by activating the κ receptor in the spinal cord, thus abridging the transmission of pain signals from the neurons to the central nervous system [53].

A possible limitation of this study is the lack of blood gas analysis, which might have resulted in an underestimation of cardiovascular function in operated donkeys. Furthermore, the duration of the surgery was rather short, necessitating further investigations to determine the suitability of the suggested anesthetic protocol for extended surgical procedures.

## 5. Conclusions

The present study concluded that the dexmedetomidine-nalbuphine combination resulted in a better antinociceptive effect and a significant reduction in postoperative pain up to 3 h post recovery when used as an adjunct to dexmedetomidine alone in Jacks undergoing field castration under general anesthesia with ketamine and propofol. Additionally, this combination led to significant cardiac improvement through the agony of nalbuphine to κ receptors. Therefore, this combination is recommended in a field situation for short-term surgical procedures. However, it is important to emphasize the use of nalbuphine in cases of hepatic dysfunction.

## Figures and Tables

**Table 1:** Results of the sedation quality, induction quality, the quality of analgesia, muscle relaxation, palpebral reflex, anesthetic maintenance, and the quality of recovery scores for dexmedetomidine (Group D) and dexmedetomidine-nalbuphine (Group DN). Results are described as median ± IQR.

Parameters	Group D (*n* = 6)	Group DN (*n* = 6)	*p*-Value
Sedation quality	(2.00 ± 0.00)	(3.00 ± 0.75)	0.022 *
Induction quality	(2.00 ± 0.75)	(1.00 ± 0.00)	0.11
Quality of analgesia	(2.00 ± 0.75)	(1.00 ± 0.75)	0.2
Muscle relaxation	(1.00 ± 0.75)	(0.50 ± 1.00)	0.5
Palpebral reflex	(1.00 ± 0.75)	(1.00 ± 0.75)	0.5
Anesthetic maintenance	(2.00 ± 0.75)	(3.00 ± 0.00)	0.11
Quality of recovery	(2.00 ± 0.75)	(1.00 ± 0.75)	0.018 *

* Indicates *p* < 0.05.

**Table 2:** Results of time for induction, duration of anesthesia, the surgical time, time to sternal recumbency, and total recovery time for dexmedetomidine (Group D) and dexmedetomidine-nalbuphine (Group DN). Results are described as Mean ± SD.

Parameters	Group D (*n* = 6)	Group DN (*n* = 6)	*p*-Value
Time for induction (seconds)	(46.83 ± 5.08)	(40.17 ± 9.33)	0.155
Duration of anesthesia (minutes)	(24.33 ± 1.75)	(23.83 ± 3.97)	0.78
The surgical time (minutes)	(16.83 ± 2.79)	(16.67 ± 3.20)	0.925
Time to sternal recumbency (minutes)	(17.00 ± 4.60)	(22.33 ± 3.01)	0.039 *
Total recovery time (minutes)	(19.83 ± 4.31)	(25.67 ± 3.14)	0.023 *

* Indicates *p* < 0.05.

**Table 3:** Effect of dexmedetomidine (D group) or dexmedetomidine-nalbuphine combination (DN group) on heart rate for jacks undergoing surgical castration using a linear mixed effects model.

Fixed Effects	Odds Ratio	95% CI	*p*-Value
Intercept	51.59	47.29–55.89	<0.001
DN	−0.67	−6.72–5.39	0.829
T (pre)	−7.76	−11.05–−4.47	<0.001
T5	−5.03	−8.10–−1.97	0.001
T15	−5.28	−8.53–−2.03	0.002
T 30	−7.56	−10.65–−4.46	<0.001
T (recovery)	−1.34	−4.69–2.01	0.431
PR ½ hour	−0.69	−3.73–2.34	0.654
DN × T (pre)	4.66	0.07–9.26	0.047
DN × T5	0.67	−3.64–4.97	0.760
DN × T15	8.02	3.45–12.58	0.001
DN × T 30	6.08	1.73–10.42	0.006
DN × T (recovery)	2.09	−2.61–6.79	0.383
DN × PR½ hour	0.43	−3.84–4.69	0.844
Random Effects			
σ^2^	24.47		
τ_00_ group	3.41		
ICC	0.12		
Marginal R^2^/Conditional R^2^	0.229/0.323		

T (pre): 10 min following premedication; T5, T15, and T30: 5, 15, and 30 min following the induction of general anesthesia, respectively; T (recovery): recovery time; PR½: ½ hour after recovery; CI: confidence intervals; and ICC: intra-class correlation.

**Table 4:** Effect of dexmedetomidine (D group) or dexmedetomidine-nalbuphine combination (DN group) on systolic and diastolic blood pressure (BP) for jacks undergoing surgical castration using a linear mixed effects model.

Predictors	Systolic Arterial BP			Diastolic Arterial BP		
Fixed Effects	Odds Ratio	95% CI	*p*-Value	Odds Ratio	95% CI	*p*-Value
Intercept	143.00	130.07–155.93	<0.001	112.00	101.56–122.44	<0.001
DN	0.67	−17.62–18.95	0.940	−1.67	−16.43–13.10	0.817
T (pre)	−56.00	−67.99–−44.01	<0.001	−46.67	−57.44–−35.89	<0.001
T5	−41.67	−53.66–−29.67	<0.001	−39.00	−49.77–−28.23	<0.001
T15	−40.33	−52.33–−28.34	<0.001	−36.33	−47.11–−25.56	<0.001
T 30	−26.00	−37.99–−14.01	<0.001	−38.33	−49.11–−27.56	<0.001
T (recovery)	−22.67	−34.66–−10.67	0.001	−20.00	−30.77–−9.23	0.001
DN × T (pre)	15.67	−1.29–32.63	0.069	15.00	−0.23–30.23	0.053
DN × T5	9.00	−7.96–25.96	0.283	19.33	4.10–34.57	0.015
DN × T15	19.67	2.71–36.63	0.025	15.00	−0.23–30.23	0.053
DN × T 30	21.33	4.37–38.29	0.016	38.33	23.10–53.57	<0.001
DN × T (recovery)	8.33	−8.63–25.29	0.319	21.33	6.10–36.57	0.008
Random Effects						
σ^2^	50.17			40.47		
τ_00_ group	22.16			11.85		
ICC	0.31			0.23		
Marginal R^2^/Conditional R^2^	0.811/0.869			0.843/0.879		

T (pre): 10 min following premedication; T5, T15, and T30: 5, 15, and 30 min following the induction of general anesthesia, respectively; T (recovery): recovery time; CI: confidence intervals; and ICC: intra-class correlation.

**Table 5:** Effect of dexmedetomidine (D group) or dexmedetomidine-nalbuphine combination (DN group) on ECG variables for jacks undergoing surgical castration using a linear mixed effects model.

Predictors	PR Interval (ms)			QT Interval (ms)			QRS Width (ms)			ST Segment Deviation (mV)		
Fixed Effects	Odds Ratio	95% CI	*p*-Value	Odds Ratio	95% CI	*p*-Value	Odds Ratio	95% CI	*p*-Value	Odds Ratio	95% CI	*p*-Value
Intercept	149.75	120.03–179.48	<0.001	344.76	263.97–425.56	<0.001	52.56	34.03–71.09	<0.001	0.00	−0.17–0.17	0.992
DN	54.11	15.40–92.82	0.006	59	−61.90–157.09	0.393	−6.17	−31.89–19.56	0.637	−0.00	−0.24–0.24	0.997
T (pre)	107.30	67.25–147.34	<0.001	216.85	137.03–296.67	<0.001	14.78	2.64–26.93	0.017	0.00	−0.15–0.15	0.999
T5	116.17	79.38–152.96	<0.001	267.98	194.65–341.32	<0.001	18.64	7.48–29.80	0.001	0.05	−0.09–0.18	0.484
T15	100.58	63.79–137.37	<0.001	147.97	74.63–221.31	<0.001	23.39	12.23–34.55	<0.001	0.07	−0.07–0.20	0.333
T 30	85.54	48.10–122.98	<0.001	217.79	143.16–292.42	<0.001	13.12	1.77–24.48	0.024	0.11	−0.02–0.25	0.107
T (recovery)	77.93	41.14–114.72	<0.001	191.36	118.03–264.70	<0.001	12.00	0.84–23.16	0.035	0.49	0.36–0.63	<0.001
PR ½ hour	10.91	−25.88–47.70	0.560	97.28	23.94–170.61	0.010	1.77	−9.39–12.93	0.755	0.00	−0.13–0.14	0.979
DN × T (pre)	−66.51	−117.00–−16.01	0.010	−145.44	−246.09–−44.79	0.005	−10.03	−25.35–5.28	0.198	0.00	−0.18–0.19	0.983
DN × T5	−116.35	−163.20–−69.51	<0.001	−202.39	−295.76–−109.02	<0.001	−0.03	−0.21–0.14	0.690	−19.97	−34.18–−5.77	0.006
DN × T15	−92.22	−140.17–−44.26	<0.001	−105.87	−201.47–−10.28	0.030	−0.07	−0.24–0.11	0.459	−22.94	−37.49–−8.39	0.002
DN × T30	−87.70	−135.89–−39.51	<0.001	−132.54	−228.60–−36.48	0.007	−0.11	−0.29–0.06	0.213	−12.46	−27.08–2.16	0.094
DN × T (recovery)	−98.77	−145.80–−51.75	<0.001	−139.61	−233.35–−45.87	0.004	−11.63	−25.90–2.63	0.109	−0.44	−0.61–−0.27	<0.001
DN × PR½ hour	−15.16	−61.84–31.51	0.523	−93.13	−186.16–−0.09	0.050	−2.18	−16.33–11.98	0.762	0.01	−0.16–0.18	0.903
Random Effects												
σ^2^	2251.58			8946.36			207.15			0.03		
τ_00_ group	39.80			935.01			71.10			0.01		
ICC	0.02			0.09			0.26			0.15		
Marginal R^2^/Conditional R^2^	0.293/0.306			0.309/0.374			0.267/0.454			0.277/0.385		

T (pre): 10 min following premedication; T5, T15, and T30: 5, 15, and 30 min following the induction of general anesthesia, respectively; T (recovery): recovery time; PR½: ½ hour after recovery; CI: confidence intervals; and ICC: intra-class correlation.

**Table 6:** Effect of dexmedetomidine (D group) or dexmedetomidine-nalbuphine combination (DN group) on respiratory rate (RR), oxygen saturation (SpO_2_%), and rectal temperature (RT) variables for jacks undergoing surgical castration using a linear mixed effects model.

Predictors	RR (Breaths/Minute)			SpO_2_%			RT (°C)		
Fixed Effects	Odds Ratio	95% CI	*p*-Value	Odds Ratio	95% CI	*p*-Value	Odds Ratio	95% CI	*p*-Value
Intercept	25.00	22.84–27.16	<0.001	92.00	89.58–94.42	<0.001	36.98	36.55–37.42	<0.001
DN	−0.67	−3.72–2.38	0.663	0.83	−2.59–4.25	0.626	−0.05	−0.67–0.57	0.872
T (pre)	−2.50	−5.38–0.38	0.088	−2.17	−5.37–1.04	0.180	−0.27	−0.88–0.34	0.385
T5	−4.17	−7.05–−1.29	0.005	−2.83	−6.04–0.37	0.082	−0.57	−1.18–0.04	0.068
T15	−5.50	−8.38–−2.62	<0.001	−3.00	−6.21–0.21	0.066	−0.78	−1.39–−0.17	0.013
T30	−4.00	−6.88–−1.12	0.007	−2.33	−5.54–0.87	0.150	−0.87	−1.48–−0.26	0.006
T (recovery)	−2.50	−5.38–0.38	0.088	−2.50	−5.71–0.71	0.123	−0.57	−1.18–0.04	0.068
DN × T (pre)	−0.67	−4.74–3.41	0.745	−0.17	−4.70–4.37	0.941	−0.05	−0.91–0.81	0.908
DN × T5	0.83	−3.24–4.91	0.684	0.50	−4.03–5.03	0.825	0.02	−0.85–0.88	0.969
DN × T15	−0.50	−4.58–3.58	0.807	−0.17	−4.70–4.37	0.941	0.05	−0.81–0.91	0.908
DN × T30	−0.00	−4.08–4.08	1.000	0.83	−2.59–4.25	0.626	0.02	−0.85–0.88	0.969
DN × T (recovery)	−0.50	−4.58–3.58	0.807	−0.17	−4.70–4.37	0.941	−0.15	−1.01–0.71	0.729
Random Effects									
σ^2^	6.22			7.62			0.28		
τ_00_ group	0.12			0.18			0.00		
ICC	0.02			0.02			0.00		
Marginal R^2^/Conditional R^2^	0.340/0.353			0.151/0.170			0.241/0.244		

T (pre): 10 min following premedication; T5, T15, and T30: 5, 15, and 30 min following the induction of general anesthesia, respectively; T (recovery): recovery time; CI: confidence intervals, and ICC: Intra-Class Correlation.

**Table 7:** Effect of dexmedetomidine (D group) or dexmedetomidine-nalbuphine combination (DN group) on WBC count, neutrophils, and neutrophil–lymphocyte ratio variables for jacks undergoing surgical castration using a linear mixed effects model.

Predictors	WBCs (×10^3^ Cells/μL)			Neutrophils (×10^3^ Cells/μL)			NLR		
Fixed Effects	Odds Ratio	95% CI	*p*-Value	Odds Ratio	95% CI	*p*-Value	Odds Ratio	95% CI	*p*-Value
Intercept	8.72	5.56–11.89	<0.001	3.15	0.74–5.56	0.012	0.72	−0.20–1.64	0.120
DN	0.55	−3.93–5.03	0.805	0.31	−3.11–3.72	0.857	−0.02	−1.32–1.28	0.977
T (pre)	−0.33	−4.24–3.58	0.865	0.37	−1.19–1.93	0.632	0.10	−0.49–0.70	0.730
T5	−0.35	−4.26–3.56	0.858	0.37	−1.19–1.93	0.635	0.14	−0.46–0.74	0.636
T15	2.36	−1.54–6.27	0.229	2.50	0.94–4.06	0.002	0.35	−0.24–0.95	0.239
T30	3.12	−0.78–7.03	0.114	4.33	2.77–5.89	<0.001	1.42	0.82–2.01	<0.001
T (recovery)	4.38	0.48–8.29	0.029	6.03	4.47–7.59	<0.001	1.84	1.25–2.44	<0.001
PR ½	3.27	−0.63–7.18	0.098	5.30	3.74–6.86	<0.001	2.08	1.49–2.68	<0.001
PR 1	5.23	1.32–9.13	0.010	7.26	5.70–8.82	<0.001	2.89	2.30–3.49	<0.001
PR 2	4.13	0.23–8.04	0.039	6.03	4.47–7.59	<0.001	2.19	1.59–2.78	<0.001
PR 4	5.05	1.15–8.96	0.012	6.68	5.12–8.24	<0.001	1.56	0.96–2.15	<0.001
PR 6	3.69	−0.22–7.59	0.064	5.74	4.18–7.30	<0.001	2.28	1.68–2.88	<0.001
DN × T (pre)	−0.13	−5.65–5.40	0.964	−0.62	−2.83–1.59	0.572	−0.14	−0.99–0.70	0.736
DN × T5	−0.37	−5.90–5.15	0.893	−0.63	−2.83–1.58	0.570	−0.07	−0.92–0.77	0.862
DN × T15	−2.10	−7.62–3.43	0.448	−1.49	−3.70–0.72	0.180	−0.05	−0.90–0.79	0.902
DN × T30	−2.21	−7.73–3.31	0.424	−3.01	−5.22–−0.80	0.009	−0.97	−1.81–−0.12	0.026
DN× T (recovery)	−2.60	−8.12–2.93	0.348	−3.76	−5.96–−1.55	0.001	−1.22	−2.06–−0.37	0.006
DN × PR ½	−2.79	−8.32–2.73	0.313	−3.93	−6.13–−1.72	0.001	−1.57	−2.42–−0.73	0.001
DN × PR 1	−3.91	−9.43–1.62	0.161	−5.68	−7.89–−3.47	<0.001	−2.36	−3.21–−1.52	<0.001
DN × PR 2	−3.21	−8.73–2.31	0.247	−4.11	−6.32–−1.91	0.001	−1.50	−2.34–−0.65	0.001
DN × PR 4	−4.54	−10.07–0.98	0.104	−5.39	−7.60–−3.18	<0.001	−0.96	−1.81–−0.12	0.027
DN × PR 6	−1.64	−7.16–3.88	0.552	−4.25	−6.46–−2.04	<0.001	−1.81	−2.66–−0.97	<0.001
Random Effects									
σ^2^	5.62			0.90			0.13		
τ_00_ group	0.59			1.13			0.16		
ICC	0.09			0.56			0.55		
Marginal R^2^/Conditional R^2^	0.328/0.391			0.736/0.883			0.723/0.876		

T (pre): 10 min following premedication; T5, T15, and T30: 5, 15, and 30 min following the induction of general anesthesia, respectively; (T recovery): recovery time; PR½: ½ hour after recovery; PR 1: 1 h after recovery; PR 2: 2 h after recovery; PR 4: 4 h after recovery; PR 6: 6 h after recovery; CI: confidence intervals, and ICC: intra-class correlation.

**Table 8:** Effect of dexmedetomidine (D group) or dexmedetomidine-nalbuphine combination (DN group) on interleukin-6 (IL-6), tumor necrosis factor-alpha (TNF-α), and cardiac troponin I (cTn-I) variables for Jacks undergoing surgical castration using a linear mixed effects model.

Predictors	IL-6 (pg/mL)			TNF-α (pg/mL)			cTn-1 (pg/mL)		
Fixed Effects	Odds Ratio	95% CI	*p*-Value	Odds Ratio	95% CI	*p*-Value	Odds Ratio	95% CI	*p*-Value
Intercept	105.65	92.04–119.25	<0.001	607.09	562.40–651.78	<0.001	39.60	31.14–48.06	<0.001
DN	−1.06	−20.30–18.17	0.913	−0.32	−63.52–62.88	0.992	0.15	−11.81–12.11	0.980
T (pre)	−1.02	−19.27–17.24	0.912	−17.61	−73.51–38.29	0.533	2.40	−5.21–10.01	0.532
T5	0.73	−17.53–18.99	0.937	−56.63	−112.53–−0.73	0.047	1.40	−6.21–9.01	0.716
T15	62.81	43.75–81.88	<0.001	210.44	152.05–268.83	<0.001	1.40	−6.55–9.35	0.727
T30	85.84	67.58–104.09	<0.001	213.22	157.32–269.13	<0.001	−1.10	−8.71–6.51	0.775
T (recovery)	50.00	30.93–69.07	<0.001	233.24	174.85–291.63	<0.001	−3.60	−11.55–4.35	0.371
PR ½	47.70	28.63–66.77	<0.001	213.94	155.55–272.33	<0.001	−3.10	−11.05–4.85	0.440
PR 1	56.08	37.01–75.15	<0.001	244.68	186.29–303.07	<0.001	−1.60	−9.55–6.35	0.690
PR 2	47.96	28.89–67.03	<0.001	219.70	161.31–278.09	<0.001	−0.60	−8.55–7.35	0.881
PR 4	48.80	29.74–67.87	<0.001	250.67	192.28–309.07	<0.001	−1.60	−9.55–6.35	0.690
PR 6	48.71	29.64–67.78	<0.001	241.19	182.80–299.58	<0.001	−1.60	−9.55–6.35	0.690
DN × T (pre)	−0.26	−26.08–25.56	0.984	−21.54	−100.60–57.52	0.590	−8.65	−19.41–2.11	0.114
DN × T5	−12.45	−38.27–13.37	0.341	−2.40	−81.47–76.66	0.952	−6.15	−16.91–4.61	0.259
DN × T15	−13.31	−40.28–13.66	0.330	−37.71	−120.29–44.87	0.367	−12.15	−23.39–−0.91	0.034
DN × T30	−34.50	−60.31–−8.68	0.009	−52.83	−131.89–26.24	0.188	−9.32	−20.08–1.44	0.089
DN × T (recovery)	−16.59	−43.56–10.38	0.225	−53.20	−135.78–29.38	0.204	−7.15	−18.39–4.09	0.210
DN × PR ½	−13.24	−40.21–13.72	0.332	−40.54	−123.12–42.04	0.332	−4.15	−15.39–7.09	0.465
DN × PR 1	−22.11	−49.08–4.85	0.107	−52.90	−135.48–29.68	0.206	−7.65	−18.89–3.59	0.180
DN × PR 2	−14.46	−41.42–12.51	0.290	−80.43	−163.01–2.15	0.056	−6.65	−17.89–4.59	0.243
DN × PR 4	−3.74	−30.70–23.23	0.784	−66.46	−149.04–16.12	0.113	−9.15	−20.39–2.09	0.109
DN × PR 6	−6.63	−33.59–20.34	0.627	−66.38	−148.95–16.20	0.114	0.35	−10.89–11.59	0.951
Random Effects									
σ^2^	230.44			2160.89			40.01		
τ_00_ group	0.83			74.14			10.12		
ICC	0.00			0.03			0.20		
Marginal R^2^/Conditional R^2^	0.747/0.748			0.843/0.848			0.268/0.416		

T (pre): 10 min following premedication; T5, T15, and T30: 5, 15, and 30 min following the induction of general anesthesia, respectively; T (recovery): recovery time; PR½: ½ hour after recovery; PR 1: 1 h after recovery; PR 2: 2 h after recovery; PR 4: 4 h after recovery; PR 6: 6 h after recovery; CI: confidence intervals, and ICC: intra-class correlation.

**Table 9:** Effect of dexmedetomidine (D group) or dexmedetomidine-nalbuphine combination (DN group) on blood glucose levels and alanine transaminase (ALT) variables for jacks undergoing surgical castration using a linear mixed effects model.

Predictors	Glucose (mg/dL)			ALT (U/L)		
Fixed Effects	Odds Ratio	95% CI	*p*-Value	Odds Ratio	95% CI	*p*-Value
Intercept	81.60	66.50–96.70	<0.001	43.97	42.80–45.15	<0.001
DN	−0.20	−21.55–21.15	0.985	0.15	−1.51–1.81	0.858
T (pre)	4.20	−14.43–22.83	0.655	−0.22	−1.69–1.24	0.761
T5	12.80	−5.83–31.43	0.176	0.18	−1.29–1.64	0.813
T15	17.00	−1.63–35.63	0.073	−1.77	−3.30–−0.25	0.023
T30	34.23	16.39–52.07	<0.001	−0.97	−2.44–0.49	0.189
T (recovery)	25.80	7.17–44.43	0.007	0.83	−0.70–2.35	0.286
PR ½	18.40	−0.23–37.03	0.053	0.05	−1.48–1.58	0.948
PR 1	10.80	−7.83–29.43	0.253	−1.02	−2.55–0.50	0.186
PR 2	9.20	−9.43–27.83	0.329	0.15	−1.38–1.68	0.846
PR 4	5.00	−13.63–23.63	0.595	0.25	−1.28–1.78	0.746
PR 6	−2.00	−20.63–16.63	0.832	0.95	−0.58–2.48	0.220
DN × T (pre)	−1.20	−27.55–25.15	0.928	2.92	0.86–4.99	0.006
DN × T5	−10.40	−36.75–15.95	0.435	2.82	0.76–4.89	0.008
DN × T15	−9.20	−35.55–17.15	0.490	5.60	3.44–7.76	<0.001
DN × T30	−21.30	−46.53–3.93	0.097	5.85	3.78–7.92	<0.001
DN × T (recovery)	−17.40	−43.75–8.95	0.193	0.30	−1.86–2.46	0.783
DN × PR ½	−5.80	−32.15–20.55	0.663	1.02	−1.14–3.19	0.349
DN × PR 1	−2.80	−29.15–23.55	0.833	1.45	−0.71–3.61	0.186
DN × PR 2	−8.40	−34.75–17.95	0.528	1.47	−0.69–3.64	0.178
DN × PR 4	−5.60	−31.95–20.75	0.674	1.50	−0.66–3.66	0.171
DN × PR 6	0.40	−25.95–26.75	0.976	0.62	−1.54–2.79	0.567
Random Effects						
σ^2^	219.78			1.48		
τ_00_ group	13.75			0.05		
ICC	0.06			0.03		
Marginal R^2^/Conditional R^2^	0.272/0.315			0.642/0.654		

T (pre): 10 min following premedication; T5, T15, and T30: 5, 15, and 30 min following the induction of general anesthesia, respectively; T (recovery): recovery time; PR½: ½ hour after recovery; PR 1: 1 h after recovery; PR 2: 2 h after recovery; PR 4: 4 h after recovery; PR 6: 6 h after recovery; CI: confidence intervals, and ICC: intra-class correlation.

**Table 10:** Effect of dexmedetomidine (D group) or dexmedetomidine-nalbuphine combination (DN group) on simple descriptive scale (SDS) variables for Jacks underwent surgical castration using linear mixed effects model.

Fixed Effects	Odds Ratio	95% CI	*p*-Value
Intercept	0.33	−0.09–0.76	0.120
DN	0.00	−0.60–0.60	1.000
PR 1	1.17	0.59–1.74	<0.001
PR 2	1.33	0.76–1.91	<0.001
PR 3	1.33	0.76–1.91	<0.001
PR 4	1.50	0.92–2.08	<0.001
PR 6	1.83	1.26–2.41	<0.001
DN × PR 1	−1.00	−1.82–−0.18	0.017
DN × PR 2	−1.00	−1.82–−0.18	0.017
DN × PR 3	−0.83	−1.65–−0.02	0.046
DN × PR 4	−0.33	−1.15–0.48	0.418
DN × PR 6	−0.50	−1.32–0.32	0.226
Random Effects			
σ^2^	0.25		
τ_00_ group	0.00		
ICC	0.01		
Marginal R^2^/Conditional R^2^	0.604/0.609		

PR 1: 1 h after recovery; PR 2: 2 h after recovery; PR 4: 4 h after recovery; PR 6: 6 h after recovery; CI: confidence intervals; and ICC: intra-class correlation.

## Data Availability

The datasets used and/or analyzed during the current study are available from the corresponding author upon reasonable request.

## Supplementary Materials

The following supporting information can be downloaded at https://www.mdpi.com/article/10.3390/ani14172452/s1, Table S1: Effect of dexmedetomidine (D group) or dexmedetomidine-nalbuphine combination (DN group) on red blood cells (RBCs) count, hemoglobin (Hb), and packed cell volume (PCV) variables for Jacks undergoing surgical castration using a linear mixed effects model; Table S2: Effect of dexmedetomidine (D group) or dexmedetomidine-nalbuphine combination (DN group) on aspartate aminotransferase (AST) and creatinine variables for Jacks undergoing surgical castration using a linear mixed effects model.

## Appendix A

Scoring criteria for assessing the quality of sedation, quality of anesthetic induction, quality of analgesia, muscle relaxation, palpebral reflex, anesthetic maintenance, quality of recovery, and simple descriptive scale (SDS) for pain.

**Parameter**	**Score**	**Description**
Sedation quality [15]	0 (No) 1 (Mild) 2 (Moderate) 3 (Marked)	- The donkey is alert with a normal stance - The donkey shows a low head carriage, ears pointed out, and a pendulous upper lip. - The donkey’s head is lowered to the ground, and its thoracic limbs have a wide stance. - The donkey exhibits a swaying movement in its pelvic limbs, which may result in the animal almost or completely falling.
Anesthetic induction quality [17]	(1) Good (2) Acceptable (3) Poor	- The donkey sinks gently to the ground, with no head or leg movements. - The donkey takes 1–2 steps before falling on the ground, with no paddling. - The donkey develops ataxia and paddling, with considerable risk to the donkey.
Quality of analgesia [19]	1 2 3 4	- No response to surgical stimulation - Brief contractions of the abdomen or leg muscles; brief twitching or spasms. - Movement of a forelimb or hind limb - Repetitive movement of either a front or hind limb necessitates the administration of more anesthetics.
Muscle relaxation [20]	0 1 2 3	- Muscle relaxation present in trunk and limbs - Muscle twitching present in some regions of trunk and limbs - Muscle twitching present over the majority of trunk and limbs - Muscle rigidity present over the majority of trunk and limbs
Palpebral reflex [4]	0 1 2	- No reflex - Induced reflex - Spontaneous reflex
Anesthetic maintenance [21]	0 (Poor) 1 (Fair) 2 (Good) 3 (Excellent)	- Multiple IV bolus doses (0.5 mg/kg) of propofol were necessary to maintain the desired level of anesthesia throughout surgery. - Administering one or two extra bolus doses of propofol to control the donkey’s movement. - The donkey appeared to be in a light plane of anesthesia. - Smooth and deep anesthetic period
Quality of recovery [22]	1 (Excellent) 2 (Good) 3 (Fair) 4 (Poor) 5 (Very poor)	- Quiet, 1 or 2 coordinated efforts to sternal and standing positions, minimal to no ataxia once standing - Quiet, 1 or 2 slightly uncoordinated efforts to sternal and standing positions, mild ataxia once standing - Multiple quiet attempts to sternal and standing positions, mild to considerable ataxia once standing - Multiple uncoordinated attempts to sternal and standing positions - Recovery resulting in major injury
Simple descriptive scale (SDS) for pain [23].	0 1 2 3	- Normal behavior, eating, alert, interactive - A little subdued, eats intermittently, a little apprehensive about interaction - Subdued, walks stiffly, occasional snatch at food only, does not interact willingly - Very stiff gait, does not eat, glancing at abdomen, may roll, does not interact at all
