# Supplementary material for: Analgesic Effect of Dexmedetomidine-Nalbuphine Combination vs. Dexmedetomidine Alone in Donkeys Undergoing Field Castration under Total Intravenous Anesthesia
Source: Animals (Basel). 2024 Aug 23;14(17):2452. doi: 10.3390/ani14172452 (PMC11393995; doi:10.3390/ani14172452)
Supplement: Supplementary file 1 [file animals-14-02452-s001.zip › animals-3130009-supplementary.pdf]

animals

MDPI

Table S1. Effect of dexmedetomidine (D group) or dexmedetomidine−nalbuphine combination (DN group) on red blood cells (RBCs) count, hemoglobin (Hb), and packed cell volume (PCV) variables for Jacks undergoing surgical castration using a linear mixed effects model.

**Predictors**	**RBCs (×10⁶ cells/μL)**			**Hb (g/dL)**			**PCV (%)**		
**Fixed effects**	**Odds ratio**	**95% CI**	***p*−value**	**Odds ratio**	**95% CI**	***p*−value**	**Odds ratio**	**95% CI**	***p*−value**
Intercept	5.47	4.76–6.17	<0.001	9.47	8.67–10.26	<0.001	33.33	28.95–37.71	<0.001
DN	0.00	−1.00–1.00	0.995	0.03	−1.09–1.16	0.952	0.20	−5.99–6.39	0.948
T (pre)	0.15	−0.85–1.15	0.763	0.20	−0.91–1.31	0.718	1.33	−4.69–7.36	0.657
T5	0.10	−0.90–1.10	0.841	−0.05	−1.16–1.06	0.928	1.33	−4.69–7.36	0.657
T15	0.13	−0.87–1.13	0.789	0.07	−1.04–1.18	0.904	2.33	−3.69–8.36	0.439
T30	-0.20	−1.20–0.80	0.683	−0.27	−1.38–0.84	0.631	0.00	−6.02–6.02	1.000
T (recovery)	-0.34	−1.34–0.66	0.500	−0.40	−1.51–0.71	0.472	−0.77	−6.79–5.26	0.799
PR ½	-0.22	−1.22–0.78	0.659	−0.17	−1.28–0.94	0.764	0.00	−6.02–6.02	1.000
PR 1	-0.30	−1.30–0.70	0.543	−0.07	−1.18–1.04	0.904	−0.03	−6.06–5.99	0.991
PR 2	-0.07	−1.07–0.93	0.893	0.23	−0.88–1.34	0.674	0.07	−5.96–6.09	0.982
PR 4	0.18	−0.82–1.18	0.720	0.30	−0.81–1.41	0.589	1.50	−4.52–7.52	0.618
PR 6	-0.07		0.893	0.13	−0.98–1.24	0.810	0.83	−5.19–6.86	0.781
DN× T (pre)	-0.11	−1.53–1.30	0.872	−0.10	−1.67–1.47	0.898	−0.53	−9.05–7.99	0.900
DN× T5	0.03	−1.39–1.44	0.970	0.32	−1.25–1.89	0.680	0.63	−7.89–9.15	0.881
DN× T15	-0.24	−1.65–1.18	0.737	−0.17	−1.74–1.40	0.832	−1.37	−9.89–7.15	0.748
DN × T30	0.02	−1.39–1.43	0.977	−0.00	−1.57–1.57	1.000	0.90	−7.62–9.42	0.832
DN× T (recovery)	0.18	−1.23–1.60	0.795	0.23	−1.34–1.80	0.766	1.57	−6.95–10.09	0.712
DN × PR ½	0.15	−1.26–1.56	0.831	0.13	−1.44–1.70	0.865	1.63	−6.89–10.15	0.701
DN× PR 1	0.13	−1.28–1.55	0.850	0.23	−1.34–1.80	0.766	1.50	−7.02–10.02	0.724
DN× PR 2	-0.20	−1.61–1.22	0.780	−0.07	−1.64–1.50	0.932	1.40	−7.12–9.92	0.742
DN× PR 4	-0.38	−1.79–1.03	0.588	−0.40	−1.97–1.17	0.610	0.30	−8.22–8.82	0.944
DN× PR 6	-0.09	−1.50–1.32	0.898	0.03	−1.54–1.60	0.966	1.13	−7.39–9.65	0.790
Random Effects									
σ^2	0.37			0.45			13.36		
$\tau_{00\ group}$	0.00			0.00			0.26		
ICC	0.00			0.01			0.02		
Marginal R^2 / Conditional R^2	0.059/0.060			0.072/0.078			0.052/0.070		

T (pre): 10 minutes following premedication; T5, T15, and T30: 5, 15, and 30 minutes following the induction of general anesthesia, respectively; T (recovery): recovery time; PR½: ½ hour after recovery; PR 1: 1 hour after recovery; PR 2: 2 hours after recovery; PR 4: 4 hours after recovery; PR 6: 6 hours after recovery; CI: confidence intervals, and ICC: intra−class correlation.

Table S2. Effect of dexmedetomidine (D group) or dexmedetomidine−nalbuphine combination (DN group) on aspartate aminotransferase (AST) and creatinine variables for Jacks undergoing surgical castration using a linear mixed effects model.

**Predictors**	**AST (U/L)**			**Creatinine (mg/dL)**		
**Fixed effects**	**Odds ratio**	**95% CI**	***p*−value**	**Odds ratio**	**95% CI**	***p*−value**
Intercept	219.50	196.33–242.67	<0.001	1.04	0.87–1.21	<0.001
DN	0.50	−32.27–33.27	0.976	0.03	−0.21–0.26	0.824
T (pre)	−11.00	−39.60–17.60	0.447	−0.08	−0.25–0.09	0.342
T5	−0.50	−29.10–28.10	0.972	−0.12	−0.29–0.05	0.155
T15	4.00	−25.87–33.87	0.791	−0.06	−0.23–0.11	0.494
T30	−1.00	−29.60–27.60	0.945	−0.02	−0.18–0.15	0.843
T (recovery)	−1.05	−30.92–28.82	0.944	−0.00	−0.18–0.17	0.955
PR ½	−2.35	−32.22–27.52	0.876	−0.04	−0.22–0.13	0.608
PR 1	−4.05	−33.92–25.82	0.788	−0.12	−0.29–0.05	0.178
PR 2	−4.40	−34.27–25.47	0.771	−0.09	−0.26–0.08	0.306
PR 4	−5.10	−34.97–24.77	0.735	−0.07	−0.24–0.10	0.434
PR 6	−6.15	−36.02–23.72	0.684	−0.06	−0.23–0.11	0.486
DN× T (pre)	14.15	−26.30–54.60	0.489	0.16	−0.08–0.39	0.183
DN× T5	6.00	−34.45–46.45	0.769	0.17	−0.06–0.41	0.146
DN× T15	4.00	−38.24–46.24	0.851	0.12	−0.12–0.37	0.325
DN × T30	3.50	−36.95–43.95	0.864	0.02	−0.21–0.26	0.852
DN× T (recovery)	2.70	−39.54–44.94	0.899	−0.00	−0.25–0.24	0.976
DN × PR ½	3.85	−38.39–46.09	0.857	0.06	−0.19–0.30	0.643
DN× PR 1	4.35	−37.89–46.59	0.838	0.07	−0.17–0.32	0.559
DN× PR 2	2.65	−39.59–44.89	0.901	0.10	−0.15–0.35	0.421
DN× PR 4	1.60	−40.64–43.84	0.940	0.10	−0.15–0.34	0.433
DN× PR 6	4.65	−37.59–46.89	0.827	0.06	−0.19–0.30	0.642
Random Effects						
σ^2		565.50			0.02	
$\tau_{00\ group}$		22.99			0.00	
ICC		0.04			0.14	
Marginal R^2 / Conditional R^2		0.031/0.069			0.161/0.278	

T (pre): 10 minutes following premedication; T5, T15, and T30: 5, 15, and 30 minutes following the induction of general anesthesia, respectively; T (recovery): recovery time; PR½: ½ hour after recovery; PR 1: 1 hour after recovery; PR 2: 2 hours after recovery; PR 4: 4 hours after recovery; PR 6: 6 hours after recovery; CI: confidence intervals, and ICC: intra−class correlation.
